# Effect of Olive Cake and Cactus Cladodes Incorporation in Goat Kids’ Diet on the Rumen Microbial Community Profile and Meat Fatty Acid Composition

**DOI:** 10.3390/biology10121237

**Published:** 2021-11-26

**Authors:** Samira El Otmani, Youssef Chebli, Bernard Taminiau, Mouad Chentouf, Jean-Luc Hornick, Jean-François Cabaraux

**Affiliations:** 1 Regional Center of Agricultural Research of Tangier, National Institute of Agricultural Research, Avenue Ennasr, BP 415 Rabat Principale, Rabat 10090, Morocco; youssef.chebli@inra.ma (Y.C.); mouad.chentouf@inra.ma (M.C.); 2 Department of Veterinary Management of Animal Resources, University of Liège, Avenue de Cureghem 6, B43, 4000 Liège, Belgium; jlhornick@uliege.be (J.-L.H.); jfcabaraux@uliege.be (J.-F.C.); 3 Department of Food Science, Food Microbiology, University of Liège, Avenue de Cureghem 6, B42, 4000 Liège, Belgium; bernard.taminiau@uliege.be

**Keywords:** olive cake, cactus cladode, goat kid, rumen liquor, microbiota, meat fatty acid

## Abstract

**Simple Summary:**

Throughout the world, the ruminant diet is based on conventional feedstuffs, which their price constantly fluctuates, and their use presents a concurrence to human nutrition. The use of alternative feed resources seems to be a solution to reduce charges and diversify ruminants’ diet. Olive cake and cactus cladodes are two alternative feed resources that are recommended to be used in ruminant feed. However, their effect on the bacterial community of ruminants is not widely investigated. This study aims to evaluate the effect of olive cake and cactus cladodes on the ruminal microbial ecosystem and meat fatty acids of goat kids. The incorporation of these feedstuffs did not change the bacterial abundance and diversity. Goat kids’ rumen liquor seemed to be able to adapt to alternative feed resources incorporation. The introduction of olive cake and cactus cladodes slightly affect meat fatty acids without a negative effect. Thus, ruminants seem to have the ability to adapt to the alternative feed resources digestion, and their use as a feed could diversify feed and reduce feed cost.

**Abstract:**

The olive cake (OC) and the cactus cladodes (CC) are two alternative feed resources widely available in the southern Mediterranean region that could be used in ruminants’ diet. Their impact on the rumen bacterial ecosystem is unknown. This work aims to evaluate their effects on the microbial community and meat fatty acids of goat’s kids. Forty-four goat kids were divided into four groups receiving diets with conventional concentrate, or 35% OC, or 30% CC, or 15% OC, and 15% CC. After 3 months, these animals were slaughtered, and the rumen liquor and *longissimus dorsi* and *semimembranosus* muscles samples were collected. Animals receiving a control diet had rumen liquor with high acidity than test groups (*p* < 0.001). Test rumen liquor was more adapted to digest efficiently their matching diet than control liquor (*p* < 0.05). These feedstuffs did not affect rumen bacteria abundance and alpha diversity (richness, evenness, and reciprocal Simpson indexes), and these results were confirmed by beta-diversity tests (NMDS plot, HOMOVA, PERMANOVA). The test diets slightly affected the individual fatty acids of meat (*p* < 0.05) without effect on fatty acids summaries, indexes, and ratios. Thus, these alternative feed resources could take place in goat kids’ diet to diversify their feed and to reduce feed costs.

## 1. Introduction

In the harsh environment, goat (*Capra hircus*) livestock is the most dominant among ruminants due to their ability to adapt to dry conditions, resist diseases, and consume low-quality forage [1]. Goats, like all ruminants, are able to provide edible foods (meat and milk) from inedible resources for humans (forage, by-products, etc.) [2,3]. In these environments, livestock is driven in the extensive system, and its diet is based mainly on rangelands. However, the pasture forage is characterized by seasonal variability that leads to animal requirement dissatisfaction, which negatively affects livestock productivity [4]. The overuse of rangelands is one of the main drivers of ecosystem degradation that could participate in the pastoral plant species extinction [1,4,5]. To meet herds’ requirements, farmers found themselves forced to supplement grazing by conventional feedstuffs. Their use presents a competition for human food and an additional cost for herders [6]. Researches recommend the use of unconventional feed resources to diversify ruminants’ diet, valorize poor quality feed, satisfy livestock needs, and reduce feeding costs and rangeland degradation [7,8,9].

The introduction of alternative feedstuff in ruminants’ diet devoid of negative effects on production and quality of products is a challenge for animal nutrition researchers [10]. Recently, consumers became more aware of the importance of product quality [11]. As well as their environmental impact [12], some researchers tried to improve the quality of products by introducing vegetable oils or feed with a high content of secondary compounds in order to alter digestion and to improve products contents in polyunsaturated fatty acids (PUFA) [6,7,13,14,15,16,17] that are beneficial to human health [11]. The alternative feedstuffs are often characterized by their content of secondary compounds (carotenoids, essential oils, antioxidants, flavors polyphenolic compounds, tocopherols, phytosterols, peptides, etc.) that could improve the meat and milk quality [6,7,13,18].

The olive cake (OC) and cactus cladodes (CC) are two alternatives feed resources widely available in the Mediterranean basin, and their use in ruminants’ diet is recommended by many studies that evaluate their composition and their effects on animal products [8,9,19,20,21]. These studies reported most of the time lack of negative effect on production, and often the improvement of products’ quality [22,23,24,25]. However, the incorporation of OC affects milk and meat quality by increasing linoleic acid contents and improving fatty acids profile [26,27,28]. In addition, Mahouachi et al. [21] reported an effect of CC on that products’ quality that could be related to digestion and microbiota because PUFA content in animals’ products results from rumen biohydrogenation shifting. The drivers of FA biohydrogenation in the rumen are mainly bacteria from the *Butyrivibrio* genus [29]. It is known that the growth of *Butyrivibrio fibrisolvens*, one of the FA biohydrogenation bacteria, is inhibited by secondary compounds such as condensed tannins [30]. Thus, it is interesting to investigate the effect of OC and CC on the bacterial community. To our knowledge, studies evaluating the effect of these alternative feed resources on small ruminants are very limited. Previous studies on the effect of OC incorporation on the bacterial community of rumen have only been conducted upon sheep and dairy ewes [14,16,31]. However, the OC effect on the ruminal ecosystem of goat kids has never been studied before. While for CC incorporation effect on the bacterial community of rumen, no studies investigating this alternative feed resource have been already published until now. Thereby, the impact of these two unconventional feed resources on the rumen bacterial ecosystem, especially of goats, has never been investigated before. Among ruminants, the ruminal ecosystem of this species is able to degrade lignin and tannins-rich feedstuffs [15,22,32,33]. In this context, this work aims to evaluate the effect of OC and CC incorporation on ruminal bacteria diversity and meat fatty acids.

## 2. Materials and Methods

### 2.1. Animals and Diets

**Figure 1:**
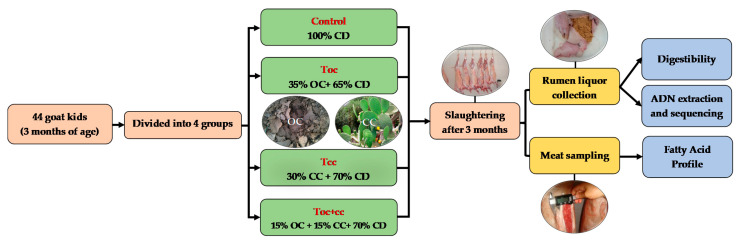
Experimental protocol scheme. CD: conventional concentrate diet (barley and faba bean); OC: olive cake; CC: cactus cladodes. T_OC_: CD with 35% of olive cake; T_CC_: CD with 30% of cactus cladodes; T_OC+CC_: CD with 15% of olive cake and 15% of cactus cladodes.

This experiment was performed in the experimental farm of the Regional Center of Agricultural Research of Tangier (INRA-Morocco) that is located at 35°67′ N and 5°85′ W. On this farm, the goat herd is of Beni Arouss Breed. This is a North Moroccan indigenous breed, well adapted to the local conditions. It is characterized by a red coat and an average bodyweight of about 42 and 38 kg for males and females, respectively [34], and an average milk production of about 600 mL/day during a lactation period of three months [35]. In the experiment, forty-four male goat kids of Beni Arouss, with an initial body weight of 10.5 ± 0.1 kg, aged three months were divided into four groups on a bodyweight basis and individually housed. During the three months of the experiment, all groups received oat hay complemented by a concentrate diet. As shown in Table 1, which presents the distributed diets and their chemical composition and nutritive value, the control group received a conventional concentrate based on barley as a source of energy and faba bean as a nitrogen source, as used by the local breeders. The other test groups received a concentrate with OC and/or CC of *Opuntia ficus indica* in addition to faba beans. The concentrate of the first test group (T_oc_) contained 35% of OC, and 30% of CC was incorporated in the second test group (T_cc_) diet. The last group (T_oc+cc_) had an administration of 15% of both (Figure 1). The OC was obtained from olive oil mills that used mechanical press process, located in Ouazzane town in Northern Morocco during the period of oil extraction (November to January). The crude OC was dried under a plastic greenhouse and conserved in plastic bags in hermetic conditions until the experiment. As for CC, they were provided daily from shrubs nearly installed. The spines of CC were manually removed, and cladodes were manually cut to slides of 9 cm^2^. The ingredients of the diet were ground and mixed to be distributed two times per day. The offered rations were adapted to allow 10% of orts that were quantified daily to determine the dry matter intake (DMI) as described by Palmieri et al. [36].

### 2.2. Slaughter and Sampling

After 3 months of the experiment, the animals were slaughtered, as detailed by El Otmani et al. [9]. The use of the animals and the experimental procedure were approved by the Regional Center of Agricultural Research of Tangier, Morocco (permit number 01/CRRAT/2017). After slaughter, rumen liquor was collected and sieved using a double cheese filter. Immediately, the rumen liquor pH was measured using a pH meter pen (HANNA HI 98120), and 1 L of rumen liquor of each group of animals was conducted in a pre-warmed thermos at 40 °C to the laboratory to be used in diets digestibility. For DNA extraction, 100 mL of each animal liquor sample was conserved at −80 °C. In addition, the longissimus dorsi and semimembranosus muscles of each carcass were removed to determine the fatty acids of goat meat.

### 2.3. Diets’ In Vitro Digestibility

The efficiency of groups of rumen liquor was evaluated by comparing the in vitro digestibility of dry (IVDMD) and organic matter (IVOMD) of control and test diets using control and test groups of rumen liquor. The IVDMD and IVOMD were performed using Ankom Daisy II Incubator^®^ (ANKOM Technology, Fairport, NY, USA) as described by Mabjeesh et al. [37]. Briefly, filter bags with 500 mg of diet samples were incubated in rumen inoculums and buffer (1:4 ratio) at 39 °C in anaerobic conditions for 48 h. In the control group, rumen liquor, all experimental diets were incubated; however, in each test group, rumen liquor, control diet, and the corresponding liquor diet were incubated. In the end, the residual DM and OM were quantified to determine IVDMD and IVOMD.

### 2.4. Microbiota Analysis

#### 2.4.1. DNA Extraction

Before DNA extraction, rumen liquor was freeze-dried using benchtop freeze dryer OPERON FDB-5502. The DNA extraction was performed according to Yu and Morrison [38]. Briefly, cell lysis was performed on 0.25 g of samples using a lysis buffer (500 mM of NaCl, 50 mM of Tris-HCl, pH 8, EDTA 50 mM, sodium dodecyl sulfate at 4% SDS) and zirconia beads (0.1 and 0.5 mm) at 70 °C during 15 min and centrifugation at 4 °C. The procedure of lysis was performed two times, and the supernatants were recuperated. The precipitation of nucleic acids was realized with 10 M ammonium acetate and isopropanol. Nucleic acids were washed with 70% of ethanol and dissolved in 100 µL of Tris-EDTA buffer (TE, Tris 50 mM, EDTA 50 mM). The DNA was purified, and RNA and proteins were eliminated by incubation with DNase-free RNase and proteinase K and using a Qiagen extraction kit (QIAamp DNA Micro Kit 50). The extracted DNA was conserved at −20 °C. To verify the quality of DNA, an electrophorese was performed on 1.2% agarose gel using Tris Borate EDTA buffer (89 mM Tris, 89 mM boric acid, 2 mM EDTA, TBE 10X pH 8.3) and a fluorescent colorant (Bioline 5X DNA loading buffer) with a horizontal box (Enduro Electrophoresis Systems—Labnet International, Inc. E1010-10) in 70 V during 40 min. The DNA visualization was carried out by Vilbert Lourmat TM Ebox TM VX2 imagins system.

#### 2.4.2. 16S rRNA Gene Library Construction and Sequencing

The V1-V3 region of 16S rDNA of each sample was amplified using (5′-GAGAGTTTGATYMTGGCTCAG-3′) and reverse (5′-ACCGCGGCTGCTGGCAC-3′) primers with Illumina overhand adapters before purification with the Agencourt AMPure XP beads kit (Beckman Coulter, Pasadena, CA, USA). For indexing, a second PCR round was performed with the primers 1 and 2 of the Nextera XT index. After purification, Quant-IT PicoGreen (Thermo Fisher Scientific, Waltham, MA, USA) was used to quantify each sample, and finale quantification was carried out with KAPA SYBR^®^ FAST qPCR Kit (KapaBiosystems, Wilmington, NC, USA). The PCR products were normalized, pooled, and sequenced by MiSeq sequencer using v3 reagents (ILLUMINA, San Diego, CA, USA). The MOTHUR software package v1.39.5 [39,40] was used to align, classify and cluster sequences into operation taxonomic unit (OTU) (average neighbor algorithm) and VSEARCH algorithm [41] to and detect and remove chimera sequences. The SILVA database (v1.32) was used as a reference for full-length 16S rDNA sequences alignment and taxonomical assignation [42]. For microbiota structure and composition analysis, a subsamples table with 10,000 sequences/samples were obtained.

### 2.5. Meat Fatty Acid

Samples of longissimus dorsi and semimembranosus muscles were collected directly after slaughter and ground to determine the fatty acids of goat meat, according to Mioč et al. [43]. The intramuscular fat was extracted using chloroform-methanol according to Folch et al. [44] method. The fatty acids methyl esters were prepared using the AOAC [45] method before their conservation in a −80 °C freezer. These fatty acids were identified by injecting in GC (Varian GC CP 3800) equipped with a flame ionization detector and a capillary column type CP-SIL88 capillary column (100 m × 0.25 mm × 0.2 µm), and compared to a standard analytical mixture of C4 to C24 FA (FAME Sigma-Aldrich, St. Louis, MO, USA) referring to 37 FA.

### 2.6. Data Analysis

The meat fatty acids were compared according to a mixed model, including the random effect animal (PROC MIXED; SAS 9.4). The effects of diet on liquor pH and of rumen liquor on diets digestibility were tested by one-way analysis of variance (ANOVA).

Bacterial data were summarized by phylum and genus, and alpha and beta diversity were determined. The studied parameters were Good’s coverage estimator, observed species, Chao1 richness index, reciprocal Simpson index, and Simpson evenness index at the genus and species levels using MOTHUR.

The effect of diet on different bacterial community abundance was estimated with a non-parametric Kruskal–Wallis H tests corrected with a Storey false discovery rate followed by Tukey–Kramer post-hoc test (STAMP 2.1.3 software). Multiple comparisons of the means were performed when a significant effect was obtained at p-value less than 0.05 using the Dunnett test.

Nonmetric multidimensional scaling (nMDS) plot, homogeneity of molecular variance (HOMOVA), and permutational multivariate analysis of variance (PERMANOVA) using Bray–Curtis dissimilarity matrix and permutation of 10,000 were performed to evaluate beta diversity.

In the case of a significant effect was obtained at *p*-value < 0.05, means were compared using Tukey’s test.

## 3. Results

The dry matter intake was variable between 854 and 1016 kg/day. This parameter was statistically similar for all groups with an average of 938 kg/day (*p* = 0.30).

**Figure 2:**
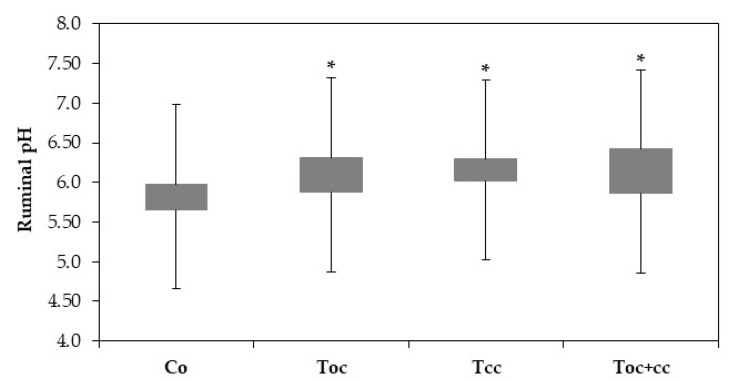
Ruminal pH of goat kids according to diets. Co: control; T_OC_: 35% olive cake; T_CC_: 30% cactus cladodes; T_OC+CC_: 15% olive cake + 15% cactus cladodes; *: groups with * are significantly different from the Co group at *p* < 0.05.

The rumen liquor pH of goat kids was very highly affected by diet (*p* < 0.001). With pH 5.7, the control liquor was more acid than all test groups (Figure 2). Other test groups’ liquor had a pH of 6.09, 6.22, and 6.21, respectively, for T_oc_, T_cc_, and T_oc+cc_.

The IVDDM and IVDOM of the control diet were the same using the control and the test groups rumen liquor (Table 2). However, test diets were more digestible by their own rumen liquor compared to the control (*p* < 0.05). The IVDDM and IVDOM of T_oc+cc_ were higher with its inoculum than control in a very highly significant way (*p* < 0.001).

**Figure 3:**
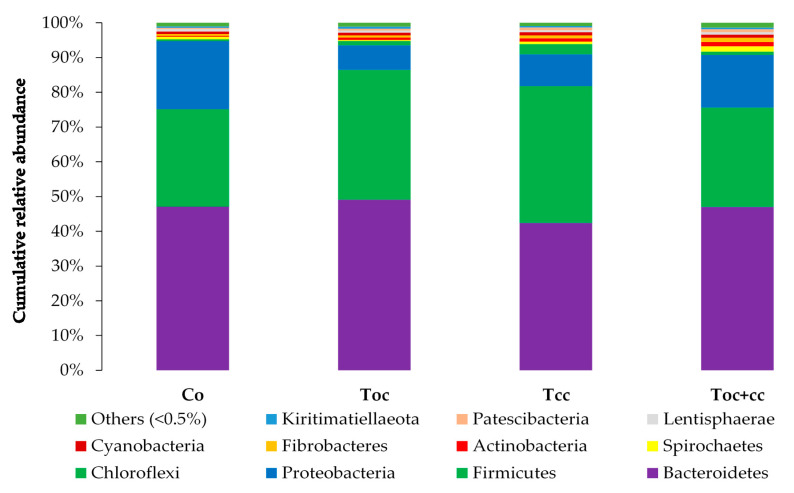
Cumulative relative abundance of bacterial community phylum according to distributed diet. Co: control; T_oc_: 35% olive cake; T_cc_: 30% cactus cladodes; T_oc+cc_: 15% olive cake + 15% cactus cladodes.

**Figure 4:**
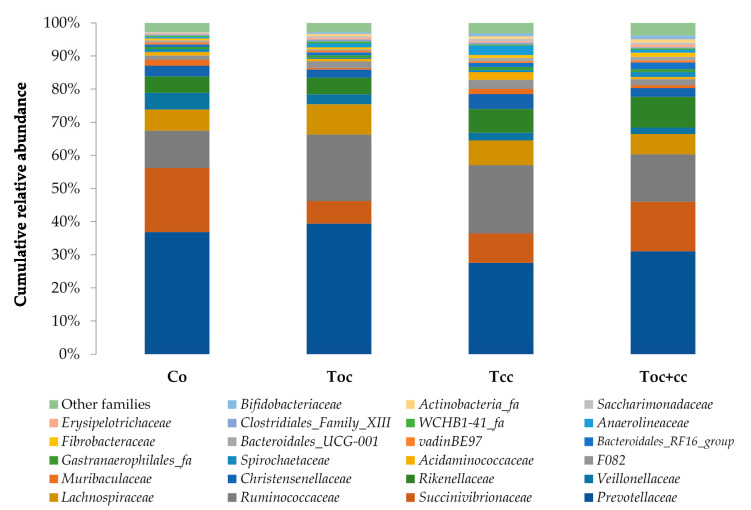
Cumulative relative abundance of bacterial community families according to distributed diet. Co: control; T_oc_: 35% olive cake; T_cc_: 30% cactus cladodes; T_oc+cc_: 15% olive cake + 15% cactus cladodes.

**Figure 5:**
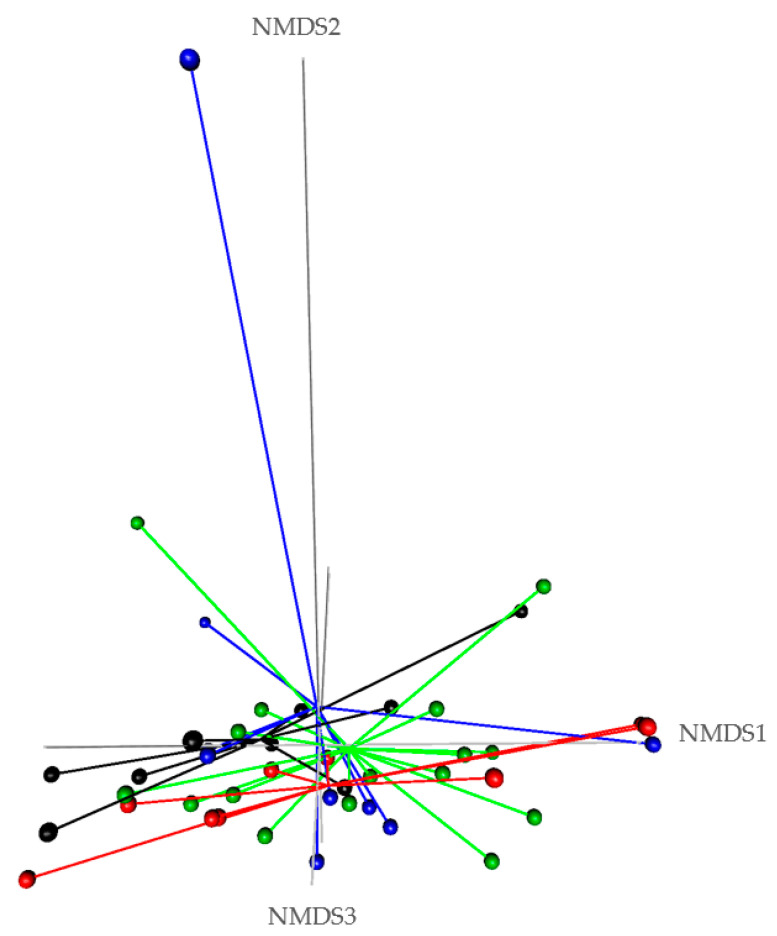
Comparison of beta diversity of rumen liquor bacterial community according to diet. Co: control; T_oc_: 35% olive cake; T_cc_: 30% cactus cladodes; T_oc+cc_: 15% olive cake + 15% cactus cladodes) using nonmetric multidimensional scaling (NMDS) plots generated by Bray–Curtis distances. (• black dot: Co; • red dot: T_oc_; • green dot: T_cc_;  • blue dot: T_oc+cc_).

From the normalized OTU table, it has been revealed 21 phyla, 38 classes, 78 orders, 125 families, 278 genera, and 8088 species of bacteria in the rumen liquor. Table 3 presents the studied parameters of alpha diversity at the genus and species levels. The sequencing of rumen liquor permitted to identify 99.8% and 93.9% of the nucleotide sequence in all groups at genus and species levels, respectively. The alpha-diversity parameters were similar in all rumen liquors. With each diet, observed species were stable with an average of 116.5 genera and 1254 species. The richness estimated by the Chao1 index did not differ in the test groups compared to the control. The evenness deduced by the Simpson index was similar in all groups with 0.06 in the control and T_oc_, and 0.07 in T_cc_ and T_oc+cc_ groups at the genus level and 0.021, 0.034, 0.047, and 0.042, respectively, in the control group, T_oc_, T_cc_, and T_oc+cc_ at the species level. No significant differences were observed between groups for the inverse Simpson index at the genus and species levels. Bacterial community composition was evaluated at three taxonomic levels. At the phylum level, the most dominant phyla were Bacteriodetes, Firmicutes, and Proteobacteria (Figure 3). Bacteriodetes abundance ranged from 42% in T_cc_ to 49% in T_oc_ liquor. The rumen liquor of Co and T_oc+cc_ contained 47% of Bacteriodetes. The Firmicutes presented an average of 33% of phyla in goat kids rumen liquor. The abundance of Proteobacteria was 7%, 9%, 15%, and 20% in T_oc_, T_cc_, T_oc+cc_, and Co liquor, respectively. Even their numerical variation, distributed diets were without an effect on the abundance of all phyla (Table 3). However, the incorporation of 35% OC and 30% CC trend to reduce the Proteobacteria phylum compared to control (*p* < 0.1). The Firmicutes/Bacteriodetes ratio was similar between groups even the average variability (1.26, 7.29, 1.58, and 4.56 in Co, T_oc_, T_cc_, and T_oc+cc_, respectively). At the bacterial family’s level, the most dominant microbiotas were from five families that were *Prevotellaceae*, *Ruminococcaceae*, *Succinivibrionaceae*, *Lachnospiraceae*, and *Rikenellaceae* (Figure 4). The abundance of *Prevotellaceae* ranged between 39% in T_oc_ and 28% in T_cc_. The family of *Ruminococcaceae* was 11%, 14%, 20%, and 21% in the liquor of Co, T_oc+cc_, T_oc_, and T_cc_, respectively. The *Succinivibrionaceae*, *Lachnospiraceae*, and *Rikenellaceae* accounted for 12.5%, 7.3%, and 6.6% of total bacterial abundance, respectively. Despite the variable values of the dominant families’ abundance, they were similar in all groups. However, the richness of *Defluviitaleaceae* was significantly increased by the administration of 30% CC (*p* < 0.05). The *Fusobacteriaceae* and *NED5E9 fa* tended to be affected by diet (*p* < 0.1). The *Fusobacteriaceae* was significantly higher in T_oc+cc_ (*p* < 0.05). The administration of CC in the diet of T_cc_ and T_oc+cc_ tended to increase the importance of *NED5E9 fa* (*p* < 0.1). At the genera level, *Prevotella_1* was the most dominant genus with an average abundance of 26%. The abundance of this genus was the same in all groups (Table 4). The *Defluviitaleaceae_UCG_011*, *Aeriscardovia*, and *Lachnospiraceae_NK4B4_group* were affected by diet (*p* < 0.05). The content of *Aeriscardovia* was significantly lower in the test groups compared to the control. The incorporation of 35% OC increased *Lachnospiraceae_NK4B4_group* content. While *Defluviitaleaceae_UCG_011* had been enriched in rumen liquor by 30% CC incorporation. The distributed diets tended to affect *Butyrivibrio_2, Marvinbryantia, Howardella* genera belonging to the *Lachnospiraceae* family, *Fusobacterium*, *Atopobiaceae_ge, Selenomonas_1*, *NED5E9_ge*, and *Ruminococcaceae_UCG-001* (*p* < 0.1). Compared to control, the incorporation of 35% OC tended to increase *Butyrivibrio_2*, *Atopobiaceae*, and *Ruminococcaceae_UCG-001* and to decrease *Howardella*. However, 30% of CC administration tended to increase *Marvinbryantia, Atopobiaceae_ge, NED5E9_ge,* and *Ruminococcaceae_UCG-001* and to reduce *Howardella* and *Selenomonas_1* compared to the control. The OC and CC combination tended to increase *Fusobacterium*, *Atopobiaceae_ge*, and *NED5E9_ge* and to decrease *Howardella*, *Selenomonas_1*, and *Ruminococcaceae_UCG-001* contents. The beta diversity was evaluated using the nonmetric multidimensional scaling plot (nMDS with three axes, Figure 5), and homogeneity of molecular variance (HOMOVA), and permutational analysis of variance (PERMANOVA). According to nMDS (Figure 5), it is clear that there is a variation in each cluster of points. All clusters are overlapped with each other except for one animal of the T_oc+cc_ group, which means that all groups are similar, and the inclusion of alternative feedstuffs did not significantly affect the beta diversity. Thus, the microbiota composition was not altered by OC and CC administration, except for one sample of the T_oc+cc_ group that was projected outside. The HOMOVA (*p* = 0.514) and PERMANOVA (*F* = 1.03; *p* = 0.349) showed no difference between groups according to distributed diet.

As detailed in Table 5, the incorporation of OC in T_oc_ increased significantly undecylic (C11:0), and docosadienoic (C22:2) acids and decreased lauric (C12:0) and selacholeic (C24:1) acids (*p* < 0.05). However, OC administration tended to increase elaidic (9t-C18:1) acid (*p* < 0.1). In the intramuscular fat, C12:0 and C24:1 decreased significantly by CC introduction in the T_CC_ group (*p* < 0.05). The arachidic acid (C20:0) tended to increase, and the 9t-C18:1 and docosahexaenoic (C22:6n-3; DHA) had a tendency to decrease with CC inclusion (*p* < 0.1). The inclusion of both of the alternative feeds in the goat kids’ diet of T_oc+cc_ increased significantly C22:2 and decreased C12:0 and C24:1 (*p* < 0.05). For FA groups, they were not affected significantly by alternative feedstuffs inclusion. However, the OC incorporation with or without CC tended to increase n-3, and the CC inclusion tended to decrease PUFA (*p* < 0.1). Comparatively to FA groups, FA ratios and indexes were statistically similar in all studied animals’ groups. Nevertheless, (C18:0 + C18:1)/C16:0 index tended to be affected by OC and/or CC incorporation with a decreased tendency in the CC group (*p* < 0.1).

## 4. Discussion

The rumen has the capacity to valorize resources that are inedible for humans (forage, concentrate, by-products, etc.) to transform them into a final edible product (milk and meat), especially in areas with a harsh environment [3]. This ability is due to its microbial ecosystem richness that living in symbiosis with the ruminants, which has the capacity to degrade fibers (cellulose and hemicellulose) to volatile fatty acids (acetate, butyrate, and propionate) as final metabolism products [3].

Rumen liquor pH depends on volatile fatty acids (VFA) production that is variable according to distributed diet and bacterial composition. Ruminal pH is related mainly to carbohydrates, fibers, and protein content of diet [46]. The pH of the control and 35% OC rumen liquor was lower than the average pH of goat close to 6.2, which presents a latent acidosis in these groups [46]. The pH of the control diet was more acid than the test groups because of its high content on barley that has a high acidogenic capacity compared to the other concentrates [47] due to its high content on carbohydrates. The incorporation of CC did not reduce the pH despite its high content in soluble carbohydrates that could disturb rumen fermentation conditions and induce, thereby, acidosis. The CC pH could not have been affected because CC mucilage stimulated salivation and allowed higher rumen fermentation stability [25].

The digestibility of ingested feed by ruminants depends mainly on their composition and the microbiota present in the digestive tract. The microbiota from the rumen liquor of test groups was able to digest the control diet constituents in the same efficiency as the control groups’ liquor. This similarity could be due to the control diet constituent that is present in the test groups’ diet. However, the control rumen liquor was less adapted to digest the test diets because they contained probably ingredients, which flora was not accustomed to.

The bacterial community of rumen liquor that provides nutrients to the host by the metabolic activities [3] is a complex system characterized by rapid changes with diet composition affecting ruminal pH and fermentation products [14,48]. A low pH value is a result of the high lactic acid production to the detriment of VFA because of the substitution of cellulolytic by lactic acid producers’ flora [46]. Many studies aimed to improve the ruminants’ products (meat and milk) quality, especially their lipid and fatty acids content, by changing diet composition [13,14,15,16]. The distributed diets could improve product quality by increasing PUFA due to perturbation of the ruminal ecosystem and implicitly of FA biohydrogenation. These diets are characterized by a high content of desirable fatty acids and/or antimicrobial activity such as polyphenols, oxalic acid, essential oils [14].

The rumen liquor of goat kids was characterized by the same diversity, richness, and evenness within groups at genera and species levels. In disagreement with the current results, Wang et al. [49] reported 1708 and 1641 for Chao1, 1557 and 1508 for observed species, and 0.98 for Simpson index in goat rumen, respectively with low and high nitrogen use, and Liu et al. [50] found a range of 1290–1746 OTU, 559–988 Chao1 and 0.94–0.98 Simpson index in juvenile goat rumen liquor. The main phyla in goat kids’ liquor were Bacteroidetes, Firmicutes, and Proteobacteria with an average of 46.4%, 33.4%, and 12.7%, respectively, and a total of both Bacteroidetes and Firmicutes of 79.8% of total phyla. Mannelli et al. [14] reported in the microbiota dairy ewes supplemented with OC Bacteroidetes and Firmicutes domination with 56% and 32%, respectively, and with a total of 89% for both that is higher than the current result. This difference could be explained by the low importance of Bacteroidetes in the current finding and the species compared to the literature. The current finding remained in the range reported by Liu et al. [50] for Bacteroidetes (33.6%–52.8%), Firmicutes (18.1%–35.3%), and Proteobacteria (3.29%–20.0%) of juvenile goat rumen. Overall, Firmicutes, Bacteroidetes, and Proteobacteria are the main phyla in rumen liquor of all ruminant species [48,51]. Many authors reported the positive correlation of Firmicutes/Bacteriodetes ratio with obesity for humans and animals [52,53] because of its role in adipogenesis [54,55]. This ratio was similar in all groups of rumen liquor even it was variable from 1.26 to 7.29. The inclusion of OC in goat kids’ diet did not affect alpha and beta diversity of rumen liquor comparatively to the finding of Mannelli et al. [14] with 2 and 3 phases OC, and of Cappucci et al. [56] with OC phenolic concentrate, and Mateos et al. [31] for dairy ewes. In addition, the CC incorporation did not alter rumen liquor diversity. The CC contains oxalate that is suspected of having an antimicrobial effect [10]. Belenguer et al. [57] reported a lack of oxalic acid effect on alpha diversity of rumen liquor of ewes. The similarity in all groups’ diversity could be due to their similar fiber content because the bacterial diversity is positively correlated to the fiber content [48,58].

The OTU was not mainly influenced by the diets. An effective of three taxas were affected by difference to results of Mannelli et al. [14], Pallara et al. [16], Mateos et al. [31] with OC inclusion in dairy ewes’ diet. This difference compared to literature could be explained by the studied species, the extraction method of used OC, the tested rumen liquor, and the essay duration. Goat species is known, compared to other ruminants, by its ability to valorize feeds with low protein and high lignin and tannins due to its microbiota specificity [15,22,32,33]. Mannelli et al. [14] reported some differences of 2 and 3 phases’ centrifugation OC effect on dairy ewes’ microbial community. This difference could be due to their variable content on residual oil and polyphenols [20]. In addition, fermentations reported in the literature were performed in vitro for 14 to 28 days using RUSITEC (rumen simulation technique) with rumen liquor of live animals [14,16,31]. At the families’ level, *Defluviitaleaceae* was significantly higher with 30% CC. Lin et al. [59] reported that *Defluviitaleaceae* is correlated negatively to obesity that explains the low fat in lean meat of semimembranosus of animals fed on CC found by several authors [9,21]. *Fusobacteriaceae* tended to be higher in the group receiving both of the resources. The *Fusobacteriaceae* family contained in goat kids’ rumen liquor one genus (*Fusobacterium*) that includes pathogen bacteria and butyric acid producers [60,61], which could explain the projection outside of the sample of this group in the NMDS plot. In the *Fusobacterium* genus, two species were observed in goat kids’ rumen liquor, which were *Fusobacterium_16S_OTU89845* and *Fusobacterium_JF975740*. The *NED5E9 fa* belongs to the Tenericutes phylum, which is increased by CC incorporation. However, this family is not well known and not well described in the literature. At the genera level, rumen liquor of the conventional diet contained significantly more *Aeriscardovia* than all test groups because the control group had a diet with barley, and this genus uses starch [62] to produce lactic acid [63], which explain the low ruminal pH of this group. In the *Lachnospiraceae* family, *Lachnospiraceae_NK4B4_group* was higher in 35% OC liquor. Combes et al. [64] found an increase in *Lachnospiraceae_NK4B4_group* with a low-protein diet before weaning in rabbits, and the protein of 35% OC diet was slightly lower than control. In addition, these results could be explained by the high content of OC with fibers that are necessary to cellulolytic bacteria growth [14]. *Butyrivibrio_2* is a butyrate producer genus found in rich fibers environment able to degrade structural carbohydrates, mainly hemicelluloses and xylan [65], to break down protein, and to biohydrogenate lipid by conversion linoleic to stearic acid [14,16,54]. The 35% OC incorporation tendency to enrich rumen liquor in *Butyrivibrio_2* could be an adaptation to degrade fibers that were slightly higher in the diet compared to control and to biohydrogenate the PUFA contained in OC. *Marvinbryantia* tended to be higher with 30% CC that could be one of the responsible factors of the lean meat of animals receiving CC because this genus is related negatively to weight and provide acetic acid from cellulose and methylcellulose degradation [66]. The goat kids’ rumen liquor with the conventional diet tended to produce more acetic acid, and this could be linked to the content of the *Howardella* genus that is an acetate producer [67]. The *Atopobiaceae* genus tended to extend with test diets inclusion. The family of this genus is known for the ability to produce lactic, acetic, and formic acids as a final product of glucose metabolism [68]. In the *Veillonellaceae* family, 35% OC rumen liquor did not affect *Anaerovibrio* content that is a lypolytic genus [69] contrarily to Mannelli et al. [14], who reported a decrease in *Anaerovibrio* genus in dairy ewes’ rumen with OC incorporation that is affecting PUFA biohydragenation [15]. This difference could be explained by the kind of used OC, the species and the sex of animals, and the experiment duration [14,15,16,20,22,31,32,33]. In addition, this difference compared to Mannelli et al. [14] could be caused by the diet composition that contains less concentrate for dairy animals in comparison to growing fattening animals’ diets because the high content of concentrate promotes *Anaerovibrio* growth [70]. The effect of OC on the *Anaerovibrio* genus in dairy ewes’ rumen liquor could be due to the tannins content that is toxic for fibrolytic bacteria [71] and the antimicrobial effect of olive oil [72,73]. Compared to sheep, goats exposed to tanniniferous feed have the ability to produce more saliva quantity and proline-rich salivary proteins that are capable of deactivating condensed tannins activity, which might explain the lack of tannins effect from OC on *Anaerovibrio* genus [74,75]. Thereby, goat kids could be adapted to degrade the high contents of condensed tannins and ether extract of OC. In the same family, the CC administration tended to impoverish the rumen liquor with Selenomonas_1, a propionate producer genus [76]. The low propionate production allows lowering fat deposit, which could be the cause of the lean *semimembranosus* of goat kids fed on CC [77]. The *Ruminococcaceae_UCG-001* belongs to the *Ruminococcaceae* family that is known for fibers digestibility and acetate production [78,79]. This genus tended to be higher with 35% OC because of the high content of this diet with fibers.

The inclusion of the studied alternative feed resources had a moderate effect on the fatty acid profile. This moderate variation could be a result of the similarity of microbiota diversity and the slight effect of diet on genus microbiota relative abundance. The increase in C11:0 by OC administration in T_oc_ could be explained by the presence of the residual olive oil in this by-product because Lerma-Reyes et al. [80] found an increase in C11:0 in goat milk following canola or soybean oil incorporation. The C12:0 that is considered as hypercholesterolemic fatty acid [11] decreased positively in goat meat with the studied resources administration. This reduction could be related to the C12:0 content in the diet [22]. Vera et al. [81] and Kotsampasi et al. [22] reported a C12:0 reduction in subcutaneous and intramuscular fat of lambs with OC inclusion, in agreement with the current result. The incorporation in T_oc_ and T_oc+cc_ groups of OC increased the C22:2 in intramuscular fat of chevon, as a result of elongation of C18:3n-3 and C18:3n-6 by elongase in association with Δ5 and Δ6 desaturase enzymes [82]. The 9t-C18:1 is an intermediate product of UFA biohydrogenation before being converted to stearic acid (C18:0). The trend for a higher concentration of 9t-C18:1 in T_oc_ meat could be due to the high content of OC in UFA and the trend for the high relative abundance of *Butyrivibrio* in T_oc_ rumen liquor, the bacteria the most involved in the biohydrogenation process.

## 5. Conclusions

The incorporation of olive cake and cactus cladodes in goat kids’ diet did not strongly change the bacterial composition of rumen liquor and meat quality. Microbiota from goat kids seems to be able to adapt to digest the alternatives feed resources. The composition of these resources and the final products of metabolism are responsible for the meat quality changes. Thus, olive cake and cactus cladodes are two alternatives feed resources without negative effect on microbiota compared to a conventional feed. They could take place in goat kids’ diet with a need for nitrogen enrichment because of their low proteins content. The inclusion of these feedstuffs could diversify the goat diet and reduce feed cost, and also their dependence only on the forest rangelands. The adaptation of goat ruminal microbiota could encourage the exploitation of inedible resources for humans and incorporate them in ruminants’ diet to provide edible foods. Further studies are recommended to evaluate the effect of these resources and other unconventional feedstuffs on the microbiota of dairy goats.

## Figures and Tables

**Table 1:** Ingredients, chemical, and nutritive composition of goat kids’ diets.

Item	Diet ^1^			
	Co	T_OC_	T_CC_	T_OC+CC_
**Diet ingredients (on DM basis)** ^2^				
Oat hay (g/kg TD)	508	427	466	456
Barley (g/kg CD)	390	0	0	0
Olive cake (g/kg CD)	0	350	0	150
Cactus cladodes (g/kg CD)	0	0	300	150
Faba beans (g/kg CD)	590	630	680	680
Vitamin-mineral supplement (g/kg CD)	20	20	20	20
**Chemical composition of diet**				
Dry matter (DM; g/kg CM)	885	887	292	435
Ash (g/kg DM)	47	47	77	62
Crude protein (CP; g/kg DM)	140	155	150	154
Ether extract (EE; g/kg DM)	35	60	33	44
Neutral detergent fiber (NDF; g/kg DM)	446	480	456	467
Acid detergent fiber (ADF; g/kg DM)	273	328	276	300
Metabolizable energy (ME; MJ/kg DM)	12	10	11	11
Forage unit for meat (FUMeat/kg DM)	0.8	0.7	0.7	0.7
Digestible proteins in the intestines (DPI; g/kg DM)	75	63	69	67

^1^ Co: control diet; T_OC_: diet with 35% of olive cake; T_CC_: diet with 30% of cactus cladodes; T_OC+CC_: diet with 15% of olive cake and 15% of cactus cladodes. ^2^ TD: total diet; CD: concentrate diet; CM: crude matter.

**Table 2:** In vitro dry and organic matter digestibility of control and test diets incubated in different groups’ rumen liquor.

Diet	Rumen Liquor	n	IVDMD	IVOMD
Co	Co	18	0.560	0.540
	T_OC_	6	0.596	0.575
	T_CC_	6	0.627	0.611
	T_OC+CC_	6	0.585	0.564
	SEM		0.012	0.013
	*p*-value		0.242	0.246
T_oc_	Co	10	0.459 ^b^	0.436 ^b^
	T_OC_	20	0.510 ^a^	0.489 ^a^
	SEM		0.013	0.013
	*p*-value		0.021	0.022
T_cc_	Co	10	0.571 ^b^	0.531 ^b^
	T_CC_	20	0.607 ^a^	0.571 ^a^
	SEM		0.008	0.009
	*p*-value		0.032	0.027
T_oc+cc_	Co	10	0.509 ^b^	0.477 ^b^
	T_OC+CC_	20	0.585 ^a^	0.555 ^a^
	SEM		0.009	0.009
	*p*-value		<0.001	<0.001

IVDMD: in vitro dry matter digestibility; IVOMD: in vitro organic matter digestibility; Co: control diet; T_OC_: diet with 35% of olive cake; T_CC_: diet with 30% of cactus cladodes; T_OC+CC_: diet with 15% of olive cake and 15% of cactus cladodes; SEM: standard error of mean; ^a,b^: values followed by different letters are significantly different at *p* < 0.05.

**Table 3:** Alpha diversity of microbiota genus and species according to diet.

Iterm	Co	T_OC_	T_CC_	T_OC+CC_	*p*-Value	SEM
**Genus**						
Good’s coverage estimator (%)	99.78	99.79	99.79	99.77	0.581	0.006
Observed genus	115.11	115.60	119.19	115.91	0.956	3.051
Chao1	134.10	135.65	138.70	138.75	0.964	3.625
Inverse Simpson index	7.00	6.62	9.12	9.04	0.479	0.707
Simpson evenness index	0.058	0.057	0.074	0.071	0.516	0.005
**Species**						
Good’s coverage estimator (%)	93.20	94.45	93.61	94.42	0.481	0.315
Observed species	1352	1162	1358	1144	0.464	61.8
Chao1	2353	1950	2269	1945	0.434	105.1
Inverse Simpson index	29.20	47.23	67.17	59.96	0.508	9.36
Simpson evenness index	0.021	0.034	0.047	0.043	0.393	0.0057

Co: control; T_oc_: 35% olive cake; T_cc_: 30% cactus cladodes; T_oc+cc_: 15% olive cake + 15% cactus cladodes; SEM: standard error of mean.

**Table 4:** Diet effect on the relative abundance of some bacterial community genus.

Family	Genus	Co	T_oc_	T_cc_	T_oc+cc_	SEM	*p*-Value
		n = 11	n = 11	n = 11	n = 11		
*Defluviitaleaceae*	*Defluviitaleaceae_UCG_011*	0.017 ^b^	0.027 ^ab^	0.037 ^a^	0.012 ^b^	0.005	0.015
*Lachnospiraceae*	*Butyrivibrio_2*	0.204	0.377	0.159	0.225	0.031	0.082
	*Howardella*	0.082	0.043	0.054	0.008	0.016	0.076
	*Lachnoclostridium_10*	0.023	0.032	0.058	0.022	0.008	0.246
	*Lachnospiraceae_NK4B4_group*	0.000 ^b^	0.005 ^a^	0.001 ^b^	0.000 ^b^	0.0006	0.044
	*Marvinbryantia*	0.142	0.157	0.237	0.098	0.022	0.067
*Fusobacteriaceae*	*Fusobacterium*	0.000	0.000	0.000	0.002	0.0003	0.052
*Bifidobacteriaceae*	*Aeriscardovia*	0.012 ^a^	0.000 ^b^	0.001 ^b^	0.000 ^b^	0.0016	0.044
*Atopobiaceae*	*Atopobiaceae_ge*	0.010	0.039	0.029	0.042	0.007	0.096
*Veillonellaceae*	*Anaerovibrio*	0.011	0.025	0.008	0.011	0.003	0.128
	*Selenomonas_1*	0.346	0.372	0.182	0.171	0.038	0.098
*NED5E9_fa*	*NED5E9_ge*	0.000	0.000	0.002	0.007	0.001	0.057
*Ruminococcaceae*	*Ruminococcaceae_UCG-001*	0.029	0.072	0.047	0.007	0.018	0.095

Co: control; T_oc_: 35% olive cake; T_cc_: 30% cactus cladodes; T_oc+cc_: 15% olive cake + 15% cactus cladodes; SEM: standard error of mean; ^a,b^: values followed by different letters are significantly different at *p* < 0.05.

**Table 5:** The effect of olive cake and cactus cladodes on fatty acid profile (g/100 g fat), groups (g/100 g fat), ratios, and indexes of goat kids meat.

Item	Co	T_OC_	T_CC_	T_OC+CC_	SEM	*p*-Value
N	11	11	11	11		
C4:0	0.308	0.457	0.370	0.452	0.039	0.651
C6:0	0.319 ^ab^	0.443 ^a^	0.207 ^b^	0.235 ^b^	0.026	0.011
C8:0	0.121	0.448	0.379	0.330	0.044	0.102
C10:0	0.385	0.120	0.254	0.135	0.044	0.194
C11:0	0.246 ^b^	0.692 ^a^	0.222 ^b^	0.426 ^ab^	0.057	0.025
C12:0	0.831 ^a^	0.193 ^b^	0.220 ^b^	0.216 ^b^	0.078	0.033
C13:0	0.268	0.299	0.274	0.157	0.031	0.183
C14:0	0.984	1.11	0.967	1.25	0.056	0.234
C14:1	0.514	0.601	0.474	0.676	0.035	0.212
C15:0	6.81	5.85	5.62	5.29	0.377	0.804
C15:1	0.888	0.519	0.613	0.572	0.062	0.461
C16:0	12.5	12.2	14.7	13.5	0.507	0.112
C16:1	1.62	2.20	1.80	1.43	0.136	0.356
C17:0	3.30	4.54	4.11	4.08	0.272	0.609
C17:1	1.74	1.25	1.36	1.13	0.096	0.286
C18:0	14.9	18.4	17.8	18.3	0.799	0.454
9t-C18:1	1.03	1.47	0.777	0.792	0.097	0.068
C18:1n-9	25.5	22.1	24.9	25.0	1.071	0.656
6t-C18:2	0.497	0.443	0.418	0.275	0.039	0.270
C18:2n-6	6.12	5.15	4.76	6.14	0.238	0.184
C20:0	0.430	0.381	0.764	0.276	0.081	0.059
C18:3n-6	0.407	0.337	0.944	0.337	0.110	0.080
C20:1	0.375	0.383	1.03	0.449	0.149	0.305
C18:3n-3	0.489	0.687	0.546	0.620	0.054	0.668
C21:0	1.24 ^ab^	1.93 ^a^	0.501 ^b^	0.727 ^b^	0.162	0.007
C20:2	0.547	0.777	0.433	0.508	0.082	0.615
C22:0	1.90	1.24	1.36	1.54	0.092	0.267
C20:3n-6	0.503	0.606	0.436	0.764	0.052	0.202
C22:1n-9	0.362	0.364	0.662	0.522	0.077	0.485
C20:3n-3	0.309	0.310	0.283	0.410	0.035	0.520
C20:4n-6	1.02	0.526	1.05	0.593	0.114	0.253
C23:0	4.89	6.16	5.68	5.35	0.248	0.306
C22:2	0.401 ^c^	1.05 ^a^	0.410 ^bc^	0.969 ^ab^	0.089	0.015
C24:0	1.18	1.82	1.17	1.12	0.141	0.350
C20:5n-3	0.734	1.36	1.15	1.40	0.128	0.385
C24:1	3.57 ^a^	1.26 ^b^	1.56 ^b^	1.36 ^b^	0.205	0.002
C22:6n-3	2.86	2.25	1.80	2.65	0.144	0.071
Summary						
SCFA	1.13	1.47	1.21	1.15	0.089	0.424
MCFA	2.84	2.90	2.16	2.73	0.330	0.560
LCFA	96.0	95.6	96.6	96.1	0.376	0.451
SFA	50.6	56.4	54.6	53.5	0.883	0.195
MUFA	35.2	29.8	32.5	31.4	0.866	0.520
PUFA	13.9	13.5	12.2	14.7	0.650	0.087
DFA	64.0	61.7	62.5	64.4	0.755	0.733
n-3	4.39	4.61	3.78	5.08	0.237	0.059
n-6	8.55	7.07	7.61	8.11	0.344	0.409
n-9	26.9	23.9	26.3	26.3	0.943	0.244
Ratio						
UFA/SFA	0.971	0.768	0.819	0.861	0.035	0.207
n-6/n-3	1.94	1.53	2.01	1.60	0.134	0.421
PUFA/SFA	0.275	0.240	0.224	0.274	0.019	0.303
MUFA/PUFA	2.54	2.20	2.66	2.14	0.312	0.513
Index						
AI	0.351	0.390	0.420	0.408	0.014	0.420
TI	0.796	0.966	1.05	0.931	0.060	0.619
(C18:0+C18:1)/C16:0	3.32	3.43	2.95	3.26	0.086	0.058
Δ9C14	0.343	0.350	0.329	0.351	0.016	0.922
Δ9C16	0.115	0.152	0.109	0.095	0.013	0.148
Δ9C18	0.641	0.561	0.591	0.584	0.013	0.599

Co: control diet; T_OC_: diet with 35% of olive cake; T_CC_: diet with 30% of cactus cladodes; T_OC+CC_: diet with 15% of olive cake and 15% of cactus cladodes; AI: atherogenicity index; TI: thrombogenic index; DFA: desirable fatty acids; MUFA: monounsaturated fatty acids; PUFA: polyunsaturated fatty acids; SFA: saturated fatty acids; SEM: standard error of mean; ^a,b,c^: values followed by different letters are significantly different at *p* < 0.05.

## Data Availability

The data presented in this study are available within the article.
